# Diagnosis and management of infertility due to ejaculatory duct obstruction: summary evidence

**DOI:** 10.1590/S1677-5538.IBJU.2020.0536

**Published:** 2020-12-20

**Authors:** Arnold Peter Paul Achermann, Sandro C. Esteves

**Affiliations:** 1 Universidade Estadual de Campinas - UNICAMP Departmento de Cirurgia (Disciplina de Urologia) CampinasSP Brasil Departmento de Cirurgia (Disciplina de Urologia), Universidade Estadual de Campinas - UNICAMP, Campinas, SP, Brasil; 2 ANDROFERT Centro de Referência para Reprodução Masculina Clínica de Andrologia e Reprodução Humana CampinasSP Brasil ANDROFERT, Clínica de Andrologia e Reprodução Humana, Centro de Referência para Reprodução Masculina, Campinas, SP, Brasil; 3 Urocore - Centro de Urologia e Fisioterapia Pélvica LondrinaPR Brasil Urocore - Centro de Urologia e Fisioterapia Pélvica, Londrina, PR, Brasil

## INTRODUCTION

Infertility, defined as the failure to conceive after one year of unprotected regular sexual intercourse, affects approximately 15% of couples worldwide (1). In about 50% of these couples, the male factor, alone or combined with a female factor, is contributory to the problem (2). Among the several male infertility conditions, ejaculatory duct obstruction (EDO) stands as an uncommon causative factor. However, the correct diagnosis and treatment may help the affected men to impregnate their partners naturally due to its treatable nature.

EDO's reported incidence among men seeking fertility varies between 1 and 5% (3, 4). Azoospermia (lack of sperm in the ejaculate) or severe oligozoospermia (less than 5 million/sperm per mL), associated with low volume ejaculate (<1.5mL, termed hypospermia) can be indicative of EDO (5). The typical clinical picture of bilateral and complete EDO includes an acidic semen specimen, a low volume azoospermic ejaculate, and low or absent fructose levels (5, 6). By contrast, oligo[astheno–terato] zoospermia can be found in patients with partial obstruction, in whom the ejaculate volume and fructose levels might be unremarkable. Nevertheless, both complete and partial obstructions can lead to infertility (7, 8). While some patients are completely asymptomatic, others complain of painful ejaculation or perineal pain exacerbated by ejaculation and hematospermia (3). These observations highlight the variability in clinical presentations, thus making a comprehensive workup paramount.

EDO is of particular interest for reproductive urologists as it is a potentially correctable cause of male infertility. Spermatogenesis is well-preserved in men with EDO owing to its obstructive nature, thus making it appealing to relieve the obstruction and allow these men the opportunity to impregnate their partners naturally. This review aims to update practicing urologists on the current methods for diagnosis and management of EDO. A detailed analysis of each therapeutic modality is provided, including the use of sperm retrieval and assisted reproductive technology.

### Anatomy and Etiology

Spermatozoa are produced in the seminiferous tubules under the influence of sexual hormones (testosterone and androstenedione) secreted by the interstitial cells. The epididymis is in continuity with the vas deferens, which in turn join the emerging ducts from the seminal vesicles to form the ejaculatory ducts (EDs). The EDs usually penetrate the central zone of the prostate and empty into the prostatic urethra on either side of the seminal colliculus (9). While the prostatic fluid accounts for approximately 0.5mL of the ejaculate, the seminal vesicles (SVs) produce an alkaline fluid with prostaglandins and fructose, which comprises 1.5-2.0mL (~50-80%) of the seminal fluid.

The ED derives from the Wolffian duct, like the epididymis body and tail, the vas deferens, and the seminal vesicles (SV). On the other hand, the prostate originates from the endoderm, which invaginates into its surrounding mesenchyme (10). Despite anatomic variations (11, 12), the ED usually runs obliquely for 1-2cm inside the prostate in a 75-angle degree (13).

Although the SVs and the EDs have similar histological features, with a cuboidal or pseudostratified columnar epithelium line and a middle collagenous layer, only the SVs present an inner muscular layer. Eighty percent of the SVs wall thickness consists of muscular layers (inner circular and outer longitudinal fibers) (13). The typical SV measures 4.5-5.5cm in length and 1.5cm in width (14). The proximal luminal diameter is larger than the distal counterpart and ranges from 1.7mm narrowing down to 0.3mm (12). The high accuracy of transrectal ultrasound (TRUS) to determine the dimensions of both the SVs and EDs makes this method a useful tool to investigate obstructions at the ED level. EDO should be suspected if TRUS shows an enlargement of the SVs, which can be congenital, acquired, or functional.

In 1914, the Zinner's syndrome was first described as a triad of unilateral renal agenesis, ipsilateral seminal vesicle cyst, and EDO as a consequence of a Wolffian duct abnormality (15). To date, less than 200 cases of this rare congenital abnormality have been reported in the literature. Agenesis or atresia of the ejaculatory ducts, mutations in the cystic fibrosis transmembrane regulator (CFTR) gene, and ectopic ureteral orifice opening directly into the ejaculatory duct are other examples of congenital causes of EDO. By contrast, acquired EDO may be secondary to trauma, infection/inflammation, or calculus. Lastly, a functional obstruction may occur as a consequence of spinal cord injury, pelvic surgery, post-retroperitoneal lymph node dissection, medication use, and systemic disorders (diabetes mellitus and multiple sclerosis) (3, 16).

### Diagnosis

The diagnosis of EDO includes history, physical examination, semen analysis, and imaging exams. The typical patient complains of painful ejaculation, which can be associated with hematospermia, decreased ejaculatory volume, and infertility. Other possible symptoms are decreased ejaculation force, perineal or lower back pain, chronic scrotal pain, and dysuria. EDO symptoms might suggest prostatitis or epididymitis, so it is essential to make the differential diagnosis (17). The presence of tender and indurated epididymis, scrotal swelling and erythema is indicative of epididymitis, whereas elevated PSA levels, dysuria, a painful prostate during digital rectal examination and an urinalysis with infection suggest prostatitis. Early endoscopic treatment may not only resolve the symptoms but also avoid progression to complete or bilateral ejaculatory duct obstruction (3, 5, 17).

Semen analysis plays a pivotal role in EDO diagnosis. While patients with complete EDO are azoospermic, those with partial EDO show severe oligozoospermia with decreased sperm motility. Other typical findings on the semen analysis of a patient with complete EDO includes a low volume ejaculate (<1.5mL) with a low pH (<7.2), and low (<13μmol per ejaculate) or absent fructose in the seminal fluid (6, 18). The finding of palpable vasa deferentia and SVs can help differentiate EDO from the congenital bilateral absence of vas deferens (CBAVD). The presence of an acidic and low volume azoospermic ejaculate, associated with absent seminal fructose, and palpable vas deferens is pathognomonic for the EDO diagnosis (18). However, the absence of one or more of these features cannot exclude EDO.

### Vasography

Vasography is carried out by incising or puncturing the vas, followed by the injection of a contrasting agent. The obstruction is confirmed by radiologic/fluoroscopic observation of normal vasa deferentia, enlarged seminal vesicles, and lack of contrast in the bladder and urethra. Despite being historically considered the gold standard method for EDO diagnosis, scrotal vasography has been replaced by TRUS. Even though the former may allow sperm collection for cryopreservation, is an invasive method that requires injection of a contrast agent, and there are risks of iatrogenic vasal stenosis or stricture. The TRUS high accuracy and no invasiveness have made this method the standard imaging diagnostic tool (3, 4, 19).

### Transrectal Ultrasonography (TRUS)

The endorectal 5-7MHz biplanar transducer has high accuracy in measuring the SVs and the ED internal diameter (3, 5). TRUS enables the evaluation of midline cysts, ED calcification, and hyperechoic SV calculi, all of which can obstruct the EDs (4, 10, 19, 20). EDO should be suspected when an enlarged SVs with a cross-section width greater than 1.5cm and/or an ED diameter >2.3mm are seen. Despite being currently advocated by many as the method of choice for evaluating infertile men suspected of having EDO-related obstructive azoospermia, TRUS has limitations. In one study, Purohit et al. performed TRUS and duct chromotubation followed by SV aspiration and seminal vesiculography in men suspected of having EDO (21). Of 25 patients with findings suggestive of EDO on TRUS, only 12 patients (48%) had the obstruction confirmed by SV aspiration and vesiculography. The authors concluded that if the diagnosis had been based on TRUS alone, only about half of the treated patients would have shown improvements in symptoms or semen analysis results (21).

### Seminal Vesicle Aspiration

The SV fluid can be aspirated with a 22-gauge and 7-inch long spinal needle under TRUS guidance to be analyzed for the presence of sperm (22). In normal conditions, motile sperm are not found in the SVs. However, the evidence is not unequivocal as Jarow, in an early study involving fertile men, reported that sperm could be found inside the SV after five abstinence days (23). However, most studies suggest that EDO should be suspected when more than three sperm-per-high-power microscopic fields (400x) are found. In one study, Engin et al. compared TRUS and TRUS-guided SVs aspiration for the diagnosis of EDO (24). They found that only half of the patients with obstructive findings on TRUS had sperm on SV aspiration. The authors suggested that SV aspiration should be added to the TRUS to improve its diagnostic accuracy (24). It has also been reported that viable sperm collected from the SV can be used for assisted reproductive technology (ART) (25).

### Seminal Vesicle Chromotubation

In this procedure, a 5mL diluted dye solution (e.g., indigo carmine or methylene blue) is injected into the SV after TRUS-guided SV aspiration. The dye efflux from the prostatic urethra is monitored with cystourethroscopy. Moreover, the method can be used to confirm obstruction resolution after endoscopic transurethral resection of the ED (TURED) (3, 21).

### Seminal Vesiculography

The TRUS-guided injection of a non-ionic contrast agent into the vesicles combined with fluoroscopy enables the evaluation of the ED anatomical and functional aspects. The lack of contrast within the urethra and bladder, associated with an enlarged SV, suggest obstruction (26). In about two-thirds of patients, this imaging exam provides information concerning the vas patency by assessing the retrograde vasogram (4).

### Magnetic resonance imaging (MRI)

The T2-weighted MRI findings indicative of EDO include the presence of an ED diameter larger than 2mm combined to the SV wall thickness and/or enhanced wall signal (27). However, like TRUS, MRI alone might overdiagnose EDO, thus leading to unnecessary surgery. Engin et al. compared these two imaging exams in a study involving 218 infertile men with suspected EDO (28). The authors concluded that TRUS should be considered the method of choice for the initial evaluation, whereas MRI should be reserved for doubtful TRUS exams. Moreover, MRI is more expensive than TRUS, not widely available, and it might miss calcifications.

### Manometry

Considered a refinement of SV chromotubation, the ED manometry evaluates the SV pressure with a spinal needle connected to a 3-way stopcock. Eisenberg et al. investigated the ED open pressure with this method and confirmed the relief of obstruction after TURED. In their study, the pressure decreased from 116cmH_2_O (range 80-150) to 54cmH_2_O (range 10-82) after ED resection (29).

The characteristics of methods for EDO diagnosis are summarized in Table-1.

**Table 1:** Characteristics of diagnostic methods for ejaculatory duct obstruction.

Methods	Invasive	Use of Contrast / Radiation
Vasography	Yes	Yes
TRUS	Yes	No
SV Aspiration	Yes	No
SV Chromotubation	Yes	No
Seminal Vesiculography	Yes	Yes
MRI	No	No
Manometry	Yes	No

**TRUS** = Transurethral Ultrasound; **MRI** = Magnetic Resonance Imaging; **SV** = Seminal Vesical

## SURGICAL TREATMENT MODALITIES AND OUTCOMES

### Transurethral Resection of Ejaculatory Duct (TURED)

#### Technique:

First described in 1973 by Farley and Barnes, TURED involves the use of a 24F resectoscope and an electrocautery loop to resect the EDs at the level of the verumontanum (30). A usual sign of success concerning the obstruction relief is the visualization of a milky or cloudy fluid flowing at the resection level. It is recommended to avoid cauterization and to use only cutting current to minimize potential scarring and prevent secondary ED stenosis (3, 30). This technique is still considered the gold standard treatment method. However, minor modifications have been introduced to decrease complications. The use of bipolar cautery, balloon dilatation, holmium laser, and smaller monopolar resection loop are examples of such technical advancements (31–33).

#### Outcomes:

Patency and semen quality improvement are achieved in up to 94% and 59% of cases after TURED respectively (21, 34, 35). Among men with complete EDO, 60% will have sperm return to the ejaculate; of those, approximately 38% of individuals will show semen parameters within normal ranges (19, 33). Natural pregnancy rates of 12-31% have been reported after TURED (19, 33–37).

#### Predictors of success:

TURED outcomes are directly related to EDO etiology. In a study by Netto et al. involving 14 infertile men with partial EDO subjected to TURED procedure, the authors showed that the group with congenital abnormalities had a more significant postoperative improvement in semen parameters than those with inflammatory or traumatic conditions. Likewise, pregnancy rates achieved naturally after ED resection was significantly higher in the congenital group than in the acquired EDO group (66.7% vs. 12.5%, respectively) (38). Other studies have reported that semen parameter improvements were more significant in partial than complete obstruction (34, 36).

#### Other indications:

For symptomatic non-infertile patients presenting with painful ejaculation and/or hematospermia, TURED has been shown to be effective in relieving the symptoms, albeit the data is minimal (39).

#### Complications:

The incidence of complications after TURED ranges from 4 to 26% (21, 33, 34, 36, 38). Bladder neck and external urinary sphincter damage, as well as obstructive scar at the ED orifice, have been reported and may result in frank hematuria, epididymal-orchitis, reflux of urine into the EDs, acute urinary retention, retrograde ejaculation, urinary incontinence or a secondary obstruction with persistent azoospermia. Erectile dysfunction and rectal perforation have also been reported (40). Extensive cauterization during TURED may result in scar formation; approximately 4% of patients with partial EDO and oligozoospermia progress to complete azoospermia postoperatively (35). Another concern is the possible reflux of urine through the EDs into the seminal vesicles. The patient may complain of “watery” ejaculate after TURED, and the presence of high creatinine levels in the semen confirm the diagnosis (40).

### Transutricular Seminal vesiculoscopy (TSV)

#### Technique:

In 2002, Yang et al. were the first to describe the TSV technique (37). For this, a 6 or 9F vesiculoscope is inserted in a retrograde fashion through the natural lumen of the ED or by puncture of the presumptive ED orifice, and holmium laser incision at the wall of the prostatic utricle is carried out. This method allows the urologist to identify and solve obstructions caused by stones, debris, and clots (41).

#### Outcomes:

In one study, Wang et al. followed 21 patients with partial or complete EDO who underwent TSV for one year (41). Seminal variables improved in 19 (90%) patients, and four couples (19%) achieved natural pregnancy. Likewise, Xu et al., in a study involving 22 men with EDO, found that 7 (31.8%) patients had significant semen parameter improvements, and six couples (27%) conceived naturally (42).

#### Predictors of success:

The complex ED anatomy can make TSV a challenging procedure. In 2018, Chen et al. were the first to distinguish the types of ED orifices using vesiculoscopy (43). Four types of ED orifices were described, namely, type A (clear ED orifice observed from the urethra); type B (ED orifice covered by a thin white membrane); type C (ED not visualized but successfully punctured in the presumptive location); type D (ED orifice not visualized and puncture not successful). Out of 419 cases, the authors found 8 (1.9%), 32 (7.6%), 341 (81.4%), and 38 (9.1%) cases in each type A, B, C, and D categories, respectively. The authors concluded that TURED should be the treatment of choice for cases in where the ED orifice is not identified (43).

#### Other indications:

Like TURED, TSV can be used for symptoms relief (e.g., painful ejaculation and hemospermia) in non-infertile patients. With TSV, it is possible to diagnose and treat seminal vesical stones, as well as to remove blood clots and excise strictures with holmium laser (41–43).

#### Complications:

Concerns with TSV includes the possibility of seminal vesicle perforation, erectile dysfunction, urinary reflux into the ejaculatory duct, epididymitis, stenosis, or rectourethral fistula (43). However, Xu et al. showed that dilating the ED with a 9F seminal vesiculoscopy was as effective as TURED, but with fewer complications (42).

### Balloon Dilation

#### Technique:

Jarow et al. were the first to describe the ED balloon dilation (44). In their case report, TRUS was used to guide the SV puncture. A 0.035-inch heavy-duty straight guidewire was used to advance the catheter through the occluded ED. Under urethroscope visualization, the correct positioning of a 4mm diameter balloon inside the ED was confirmed. The balloon was inflated twice to ensure adequate dilation. Subsequently, balloon dilation under CT-guidance was proposed (45).

#### Outcomes:

Only a few cases series exist, describing pelvic pain resolution, without complications, but no data concerning semen parameters improvement or pregnancy achievement exist (44, 46).

#### Predictors of success:

No study has yet compared this technique with other treatment modalities.

#### Other indications:

Although most EDO treatment modalities aim to improve semen parameters and fertility, the few cases series on balloon dilation published to date only reported chronic pelvic pain relief.

#### Complications:

Not reported.

### Midline Prostatic Cyst Aspiration

#### Technique:

Midline prostatic cysts (MPC) are found in about 10-17% and 5.8% infertile and fertile men, respectively (19, 47). Under local anesthesia and TRUS-guidance, an 18-gauge 200mm-long needle is inserted into the MPC. The fluid is aspirated with a 20mL syringe and examined at 400× magnification to verify if spermatozoa exist (47).

#### Outcomes:

In a retrospective cohort study published by Lotti et al., eleven patients with cysts >0.25mL underwent TRUS-guided cyst aspiration (TRUCA) (47). One month later, all patients had their sperm count improved. However, the improvement was temporary, and three months after the procedure, the cyst volume increased, and the sperm count declined, albeit the decline was not so remarkable to bring the semen parameters to baseline levels. After a one-year follow-up, five patients achieved pregnancy, four of them by natural intercourse, and one by intracytoplasmic sperm injection (ICSI).

#### Predictors of success:

Limited data indicate that cysts with volumes higher than 0.117mL might affect fertility (47). On this basis, the treatment of such cysts might be associated with improved outcomes.

#### Other indications:

Not reported.

#### Complications:

A temporary and self-limited hematospermia was described after TRUCA (47).

Table-2 summarizes the evidence of studies reporting treatment outcomes for EDO.

**Table 2:** Characteristics of studies reporting treatment outcomes for patients with ejaculatory duct obstruction.

Study	Reference	Design	Follow-up	Patients	Method of diagnosis	Surgical Technique	Outcomes	Predictors of success	Other indications	Complications
Farley and Barnes (1973)	30	Retrospective cohort	2 to 15 years	18 symptomatic patients (fertility not evaluated)	Clinical symptoms	TURED	All 18 patients had symptoms relieved	NR	Relief of symptoms such as perineal pain, painful ejaculation, pain in one or both groins, low back pain	Recurrence of ED obstruction in 8 patients (44%)
Jiang et al. (2014)	31	Prospective	9 to 52 months	51 patients with obstructive azoospermia (11 with EDO)	TRUS	TURED (holmium laser)	Patency achieved in 10 of 11 patients (90.9%); 4 natural pregnancies, and 1 pregnancy by IUI	NR	NR	Temporary hematospermia which resolved in 7-10 days
Yang et al. (2002)	37	Prospective	3 to 36 months	37 hematospermic patients	TRUS; MRI	TSV	NR	NR	Hematospermia & possible SV invasion from prostate carcinoma	none
Manohar et al. (2008)	39	Prospective	3 months	25 patients	TRUS; Seminal Vesiculography; SV aspiration	TUIED (hook electrode)	Relief of pain in 96% of patients; No data on fertility	NR	Relief of symptoms (hemospermia, painful ejaculation, severe perineal discomfort)	12% (3/25) Epididymo- orchitis
Chen et al. (2018)	43	Prospective	12 months	419 hematospermic patients	TRUS; MRI	TSV (381); TURED; TUIED (holmium laser)	Fertility not evaluated; Hematospermia alleviated or solved in 85% of cases (324/381) by TSV	NR	Relief of symptoms (hematospermia)	Retrograde ejaculation (TURED): 20% (1/5) Epididymitis: 0.4% (2/414)
Jarow et al. (1995)	44	Case report	NR	1 case	TRUS; Seminal Vesiculography; SV aspiration	BD	Fertility not evaluated; Pelvic pain solved	NR	Relief of symptoms (pelvic pain)	none
Lawler et al. (2006)	46	Case report	9 months	1 case	TRUS; MRI; SV aspiration; Seminal Vesiculography	BD	Fertility not evaluated; Relief of chronic pelvic pain	NR	Relief of symptoms such as pelvic pain	none
Lotti et al. (2018)	47	Prospective	12 months	66 infertile men with MPC; 582 infertile men without MPC; 103 fertile men	TRUS	TRUCA (11 patients)	Reduction of MPC volume; Natural pregnancy: 36.4% (4/11)	MPC volume > 0.117 ml associated with impaired semen quality	NR	Transient hematospermia
Tu et al. (2013)	33	Prospective	6 to 24 months	38 azoospermic and 4 severe oligozoospermic patients	TRUS; Vasography; MRI	TURED (bipolar)	Improved semen volume, pH level, and semen fructose; Sperm return to ejaculate semen in 23 of 42 cases (60.5%); All severe oligozoospermic patients had semen improvement postoperatively; Normal semen analysis in 16 of 42 patients postoperatively (38.1%); Pregnancy in 13 of 42 patients (31%) in 18 months follow-up	NR	NR	Epididymitis; Watery ejaculate; Temporary hematospermia (<3months)
Vazquez- Levin et al. (1994)	40	Prospective	1 to 3 months	8 patients	TRUS	TURED	Semen parameters improvements in 62.5% (5/8) cases; Pregnancy in 12.5% (1/8) cases	All patients had an increase in the seminal plasma creatinine levels. The only patient who did not show significant creatinine levels in the seminal plasma after TURED achieved pregnancy	n/a	Transient urinary retention
Wang et al. (2012)	41	Prospective	1 to 12 months	21 patients	TRUS	TSV; TURED (when failure to find ED orifice)	Improvements in ejaculate volume and sperm count; Relief of pain and hematospermia resolution; Natural pregnancy in 4 of 21 patients (19%)	NR	Relief of symptoms; (hematospermia and painful ejaculation)	Mild pain
Turek et al. (1996)	35	Retrospective	2 to 48 months	46 patients	TRUS	TURED	Normal postoperative semen volume in 17 of 36 patients (46%); All treated patients improved and had motile sperm in ejaculates; Pregnancy achieved in 9 of 46 patients (20%)	NR	NR	20% (10/46); One oligozoospermic patient became azoospermic after surgery; Watery ejaculate; Gross hematuria; Urinary tract infection; Chronic epididymitis; Post-void dribbling; Premature ejaculation.
El-Assmy et al. (2012)	36	Retrospective	9 to 60 months	17 patients with complete EDO and azoospermia; 6 aptients with partial EDO and oligozoospermia	TRUS; MRI	TURED	Improvements in ejaculate volume, sperm concentration, and percent motility; Natural pregnancy in 3 of 23 patients (13%)	Semen improvements: Partial EDO – 100% (6/6) Complete EDO – 23.5% (4/17) Positive sign: midline cyst	NR	26% (6/23) Epididymo- orchitis; Watery ejaculate; One oligozoospermic patien became azoospermic after surgery
Netto et al. (1998)	38	Prospective	8 month to 5 years	6 congenital partial EDO; 8 acquired partial EDO	TRUS Vasography	TURED	Pregnancy in 35.7% (6/14) of cases; Improvements in semen parameters – 64.3% (9/14) cases	Congenital group: all patients had semen parameters improvements (100% - 6/6), and 66.6% (4/6) achieved pregnancy; Acquired group: 37.5% (3/8) had semen parameters improvements, and 12.5% (1/8) achieved pregnancy	NR	Watery ejaculate; High volume ejaculate; Urinary tract infection; One oligozoospermic patient became azoospermic after surgery
Xu et al. (2011)	42	Prospective	12 months	16 partial EDO 6 complete EDO	TRUS; Vasography	TSV (18 cases); TURED (when failure to find ED orifice) 4 cases	Semen parameters improvements in 81.8% (18/22) cases; Normal postoperative semen analysis in 31.8% (7/22) cases; Pregnancy in 6 of 22 patients (27.3%)	n/a	Relief of symptoms (hemospermia, painful ejaculation, and perineal pain)	Only one case with urine reflux into SV after TURED; No complications with TSV
Kadioglu et al. (2001)	34	Prospective	12 to 63 months	22 men with azoospermia and complete EDO; 16 men with oligozoospermia and partial EDO	TRUS; MRI	TURED	Increased ejaculate volume, sperm concentration, and percent motility; Relief of symptoms, hematospermia and painful ejaculation resolution; Natural pregnancy in 5 of 13 patients (13%); 15 patients achieved sperm concentration of >5 million/ml	Semen improvements in 59% of complete EDO, and 94% of partial EDO (p=0.04); Positive signs for success: midline cyst, dilated SV, eccentric cyst; Negative sign: ED calcification	Relief of symptoms (hemospermia, painful ejaculation, and perineal or testicular pain and discomfort)	13% (5/38); Urinary tract infection; Recurrent epididymitis; Acute urinary retention One oligozoospermic patient became azoospermic after surgery

**TURED** = Transurethral Resection of Ejaculatory Duct; **TUIED** = Transurethral Incision of Ejaculatory Duct (holmium laser); **TSV** = Transutricular Seminal Vesiculoscopy; **BD** = Balloon Dilation; **MPC** = Midline Prostatic Cyst; **TRUCA** = Transrectal Ultrasonically-guided Cyst Aspiration; **n/a** = not available; **AO** = Obstructive Azoospermia; **EDO** = Ejaculatory Duct Obstruction; **ED** = Ejaculatory Duct; **SV** = Seminal Vesicles; **TRUS** = Transurethral Ultrasound; **MRI** = Magnetic Resonance Imaging; **IUI** = intrauterine insemination; **NR** = not reported

Achermann & Esteves. Summary evidence of EDO diagnosis and management. Int Braz J Urol 2021.

### Assisted reproductive technology

As discussed in previous sections, the surgical repair is a cost-effective therapy for infertile men affected by EDO-related obstructive azoospermia. However, this approach might not be feasible nor desired by the couple. Moreover, late obstruction has been reported after EDO treatment, and in some cases, the semen parameters remain suboptimal to allow natural conception (34, 35, 42). Thus, ART, in particular, ICSI, has been applied to overcome infertility in such cases (48).

Sperm injections can be carried out with ejaculated sperm or sperm retrieved from the seminal vesicles, epididymis, or testis. In the cases of partial EDO, or when postoperative semen parameters remain suboptimal, ejaculated spermatozoa can be used for ICSI. By contrast, sperm retrieval has to be done in complete EDO, including the cases in which surgical treatment has failed. EDO is characterized by the presence of normal spermatogenesis; thus, sperm retrieval is successful in virtually all cases. Percutaneous epididymal sperm aspiration (PESA), microsurgical epididymal sperm aspiration (MESA), testicular sperm aspiration (TESA), an testicular sperm extraction with or without the aid of microsurgery (micro-TESE and TESE, respectively) can be used to harvest sperm for ICSI (49). The cause of OA and the sperm retrieval technique seem to have little influence on sperm retrieval rates and ICSI outcomes (50). The reported live birth rates with ICSI range from 32 to 36% in the population of men with OA, including EDO (50).

### Management of functional EDO

Rare cases of functional EDO have been associated with spinal cord lesions, neurologic disorder (e.g., multiple sclerosis), diabetes mellitus with neuropathic changes, iatrogenic neural damage after retroperitoneal lymph nodes dissection, pelvic surgery or fracture, or medication (e.g., alpha-adrenergic blockers, antipsychotics, thiazide diuretics, and tricyclic antidepressants). Furthermore, it has been speculated that a functional obstruction can occur after TURED in patients who remain with enlarged SVs (16). In such cases, sperm retrieval and ICSI may be offered. There is an overall lack of data for other interventions to treat functional EDO, including diabetes treatment and medication cessation or substitution. Nevertheless, some authors have suggested using oral phosphodiesterase inhibitors (e.g., PDE5i) in diabetic patients with functional EDO, which might improve SV ejection fraction, seminal analysis, and fructose (16, 51).

**Figure 1:**
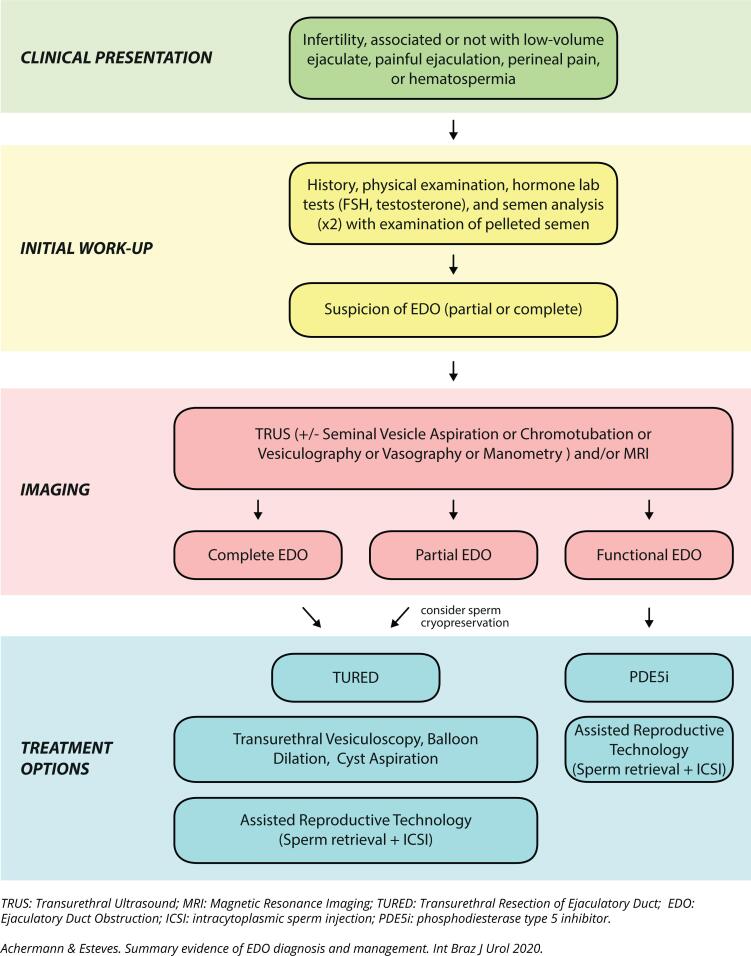
Algorithm for the diagnosis and management of infertile with with ejaculatory duct obstruction.

Figure-1 depicts an algorithm for the diagnosis and management of infertile men with EDO.

## CONCLUSIONS

EDO is an uncommon cause of infertility, with considerable variability in its clinical presentation (Figure-1). A comprehensive workup, including medical history, semen analysis, and imaging is essential for the correct diagnosis and management. Although TURED is still considered the gold standard treatment, patients should make informed decisions with their physicians after weighing the risks and benefits of each treatment modality and the intended goal. Spermatogenesis is preserved in men with EDO; thus, sperm can be easily retrieved from both the epididymis and testicles, and ICSI might be a valid alternative for couples to achieve biological parenthood.
